# One-Step In Situ Inkjet Printing Fabrication of Au-Decorated Polyaniline on MEMS Platforms for Sensitive Ammonia Sensing at ppb-Level

**DOI:** 10.3390/mi17080925

**Published:** 2026-07-31

**Authors:** Jin Zhang, Dawu Lv, Ye Yang, Weijie Song, Ruijin Yu, Wenfeng Shen

**Affiliations:** 1 Ningbo No.2 Hospital, Ningbo 315099, China; 2 Zhejiang Provincial Engineering Research Center of Energy Optoelectronic Materials and Devices, Ningbo Institute of Material Technology and Engineering, Chinese Academy of Sciences, Ningbo 315201, China; 3 Center of Materials Science and Optoelectronics Engineering, University of Chinese Academy of Sciences, Beijing 100049, China; 4 College of Chemistry & Pharmacy, Northwest A&F University, Yangling 712100, China; 5 Research and Manufacturing Base of Olfactory Sensors in China Sensing Valley, Bengbu 233040, China; 6 CS-Microsensor (Ningbo) Technology Co., Ltd., Ningbo 311121, China

**Keywords:** ammonia sensor, Au–PANI nanocomposite, inkjet printing, MEMS micro-hotplate

## Abstract

High-performance ammonia (NH_3_) sensors play a critical role in environmental protection and noninvasive medical diagnosis. This work reports a new NH_3_ sensor based on Au-microsphere-decorated polyaniline (PANI) manufactured by a precise in situ inkjet printing method on a MEMS micro-hotplate. The in situ oxidative polymerization of aniline was performed directly on the MEMS platform using AuCl_3_ as a bifunctional oxidant and precursor, with a hierarchical morphology of microspheres (~750 nm) and nanorods (~250 nm). Reduced from Au^3+^ in the polymerization reaction, Au microparticles achieve substantial catalytic promotion by virtue of chemical sensitization and spillover effect. The optimized Au–PANI MEMS sensor exhibits a superlative response of 201% toward 1 ppm NH_3_ at room temperature, with an ultra-low theoretical limit of detection (LOD) of 0.42 ppb. Furthermore, the device demonstrates rapid response/recovery kinetics (76 s/72 s), exceptional selectivity against common interfering gases (SO_2_, CO, H_2_, etc.), and robust long-term stability with high response retention over two months. This research provides a scalable, cost-effective strategy for the mass production of miniaturized, high-sensitivity gas sensors for industrial and healthcare applications.

## 1. Introduction

Ammonia (NH_3_) is a critical chemical commodity extensively utilized in industrial synthesis, agricultural fertilization, and pharmaceutical manufacturing [1]. NH_3_ is an industrially useful gas but it is highly poisonous, corrosive and irritating and can cause serious harm to human health. Acute or chronic exposure can result in irreparable damage to the respiratory and immune systems. The safety threshold is rigorously regulated at 35 ppm for an exposure period of 10 min [2]. Besides industrial safety, trace ammonia in human exhaled breath has become a crucial physiological biomarker for non-invasive diagnosis of renal failure, cirrhosis and stomach ulcers [3,4]. Consequently, the development of high-performance gas sensors capable of real-time, high-precision NH_3_ monitoring is indispensable for production safety, environmental governance, and advanced medical diagnostics.

Organic conducting polymers, specifically polyaniline (PANI), are one of the most researched platforms for gas detection because they have a relatively low manufacturing cost, good long-term environmental stability, and operate at room temperature [5,6]. Despite this interest, the use of PANI-based sensors is generally limited by the fact that pure PANI sensors have several limitations, including limited sensitivity at ppm or ppb level concentrations, slow response times and recovery rates, and high cross-sensitivities to humidity, making it difficult to deploy them in real-world applications [7]. Structural modification of PANI by incorporating gold particle structures into the PANI polymer structure has been shown to be a very effective way to modify the behavior of the PANI sensor. In particular, gold has a specific 5d^10^6s^1^ electronic configuration and a well-known catalytic activity. This results in a modification of the electronic properties of the PANI sensor [8]. Therefore, the addition of Au particles increases both the number of surface active sites on the organic conducting polymers and provides additional electronic/chemical sensitization pathways to enhance the interaction between the PANI sensor and hydrogen-containing species such as ammonia [9].

Despite the considerable progress in Au-modified PANI NH_3_ sensors, existing material and device architectures still suffer from inherent performance and fabrication trade-offs, leaving critical gaps for practical MEMS-level integration. Au-functionalized MoSe_2_/Ti_3_C_2_T_x_ MXene heterostructures achieve ppb-level sensitivity but require high-temperature operation (75 °C), exhibit poor selectivity, and are incompatible with microfabrication via conventional drop-casting [1]. p-type GaN-HP/PANI heterostructures enable room-temperature sub-ppm detection but rely on complex etching of brittle GaN substrates and suffer from slow recovery at low NH_3_ concentrations [10]. TiO_2_/PANI and other PANI–oxide composites deliver improved response speed compared with pure PANI, yet their detection limits remain at tens of ppm and cannot be miniaturized on rigid microdevices [11]. Physically blended Au nanostar/PANI composites achieve enhanced room-temperature sensitivity, but weak interfacial coupling causes degraded reproducibility, prolonged recovery, and a relatively high limit of detection (~20 ppm) [12]. A common limitation of these reported strategies is the separate synthesis of noble metal and PANI components, which impairs interfacial interaction and device uniformity. More importantly, few existing sensors can simultaneously realize room-temperature ppb-level sensitivity, scalable microfabrication, and long-term stability on a single MEMS chip.

To address the above bottlenecks, advanced microfabrication strategies compatible with MEMS platforms are urgently required to construct high-quality, miniaturized PANI-based sensing films. Inkjet printing, as a non-contact additive manufacturing technique, offers distinct advantages over conventional spin-coating and drop-casting methods. It enables high-resolution patterning, layer-by-layer material deposition, and zero mechanical damage to substrates, while effectively avoiding cross-contamination of functional layers [13,14]. Such merits make inkjet printing highly suitable for the batch fabrication of high-precision, miniaturized gas-sensing microdevices.

Based on our previous MEMS Au–PANI sensor prototype [15], which was fabricated via conventional casting and only achieved ppm-level detection, this work further advances the scalable and high-performance MEMS sensing platform via innovative inkjet printing and in situ polymerization strategies. Herein, AuCl_3_ is innovatively employed as both the oxidant and dopant for aniline polymerization, enabling one-step in situ growth of Au microsphere-decorated PANI films with tight interfacial bonding directly on MEMS micro-hotplates through sequential inkjet printing. The optimized sensor exhibits an ultra-low limit of detection of 0.42 ppb, a high response of 201% toward 1 ppm NH_3_, excellent cyclic repeatability, superior long-term stability over 70 days, and outstanding selectivity against interfering gases. This in situ inkjet printing strategy provides a facile and scalable route for high-performance trace NH_3_ sensing and offers a universal manufacturing paradigm for future wearable, intelligent, and industrial gas-sensing MEMS devices.

## 2. Experimental Section

### 2.1. Materials and Reagents

Analytical-grade chemical reagents, encompassing HCl (36–38%), AuCl_3_ (99%) aniline, citric acid, acetone, formaldehyde, methanol, toluene, and ethanol, were directly procured from Sinopharm Chemical Reagent Co., Ltd. (Shanghai, China). Polyvinylidene fluoride (PVDF) membranes and conductive silver paste came from Hangzhou Auto Experimental Consumables Co., Ltd (Hangzhou, China). Deionized (DI) water was used in all experimental procedures. All chemical reagents were used as received without further purification. Standard gas mixtures of 100 ppm NH_3_, 200 ppm SO_2_, 200 ppm H_2_, and 200 ppm CO were purchased from Zhengzhou Xingdao Chemical Technology Co., Ltd. (Zhengzhou, China), with high-purity synthetic air serving as the balance gas. Volatile organic vapors, including methanol, ethanol, acetone, toluene, and formaldehyde, were continuously generated using an Owlstone vertical vapor generator (V-OVG, Owlstone Inc.) (Westport, CT, USA). All gas-sensing measurements were performed under high-purity synthetic air as the background and carrier atmosphere. The target gases and organic vapors were diluted to desired concentrations through dynamic volumetric dilution in synthetic air prior to testing.

### 2.2. Material Characterization and Device Measurement

The morphological features and microstructures of the sensing materials were examined by field-emission scanning electron microscopy (FE-SEM, Hitachi SU8200) (Tokyo, Japan). The chemical composition, crystallographic structure and functional groups were further analyzed by several complementary techniques: X-ray photoelectron spectroscopy (XPS, Thermo Fisher K-alpha) (Hillsboro, OR, USA) X-ray diffraction (XRD, Bruker D8 ADVANCE, Cu Kα1 radiation, λ = 1.5406 Å) and Fourier-transform infrared spectroscopy (FT-IR, Thermo Nicolet 6700) (Madison, WI, USA).

Real-time electrical resistance changes in the sensors were measured using a self-built static volumetric measurement system. Resistance and other experimental data during the experiment were sent via a wireless communication link to a monitoring computer (via a Bluetooth module). Temperature and humidity within the test chamber were kept at a fixed level using a programmable constant temperature/humidity cabinet from Dohe@Test (DHTHM-27-20-p-sd) (Shanghai, China). The following formula provides a quantitative definition of the dynamic sensor response (Response):

$$Response\left(\%\right)=\frac{R_{g}-R_{a}}{R_{a}}\times 100\%$$

In this equation, R_g_ denotes the stable value of the sensors’ electrical resistances in the presence of the reference gas, whereas R_a_ signifies the stable value of the sensors’ resistances in ambient air. The response and recovery times were defined as the duration needed for the sensors to attain a maximum or minimum 90% alteration in their total sensor resistance upon reaching saturation or desorption. During all gas-sensing measurements, experimental conditions were precisely and stably controlled. The sensor operating temperature was controlled with an accuracy of ±5 °C. The target gas concentration was regulated within an error of ±0.1 ppm, and the ambient humidity was maintained at a precision of ±5% RH, guaranteeing stable and reproducible sensing performance tests. All gas-sensing measurements were conducted at a constant operating voltage of 3 V, with a fixed 5 kΩ load resistance adopted in the test circuit for signal acquisition. All electrical tests were performed at room temperature under stable DC voltage operation.

### 2.3. Preparation of Au–PANI MEMS Sensors by Inkjet Printing

The fabrication of Au–PANI MEMS sensors proceeded as follows. For ink formulation, 0.3 mmol aniline was dissolved in 10 mL of 1 M hydrochloric acid and magnetically stirred at room temperature for 2 h to prepare the aniline precursor. Separately, 0.08 mmol of AuCl_3_ was dissolved in 10 mL of 1 M hydrochloric acid solution to prepare the AuCl_3_ precursor.

These precursor inks were loaded into cartridges and deposited onto the MEMS micro-heated plate via a flexible electronic printer (MP1100, Shanghai Zhongbin Technology Co., Ltd.) (Shanghai, China). This printing system works under drop-on-demand mode, fitted with 16 functional nozzles of 20 μm diameter to eject 10 pL droplets; the drop spacing, jetting frequency and driving voltage were set to 20 μm, 5 kHz and 24 V, respectively. In the printing sequence, the aniline precursor was first printed onto the MEMS platform, followed by the AuCl_3_ precursor. As post-printing treatment, the printed sensors were stored in a refrigerator at 0–5 °C for 48 h to accomplish in situ oxidative polymerization, then dried at 100 °C for 20 min after the reaction finished.

For control experiments, additional Au–PANI MEMS sensors were fabricated with gradient concentrations of AuCl_3_ (0.001–0.012 M) and aniline (0.01–0.07 M) for comparative analysis.

## 3. Results and Discussion

### Microstructural and Physicochemical Characterization of the Sensing Materials

**Figure 1:**
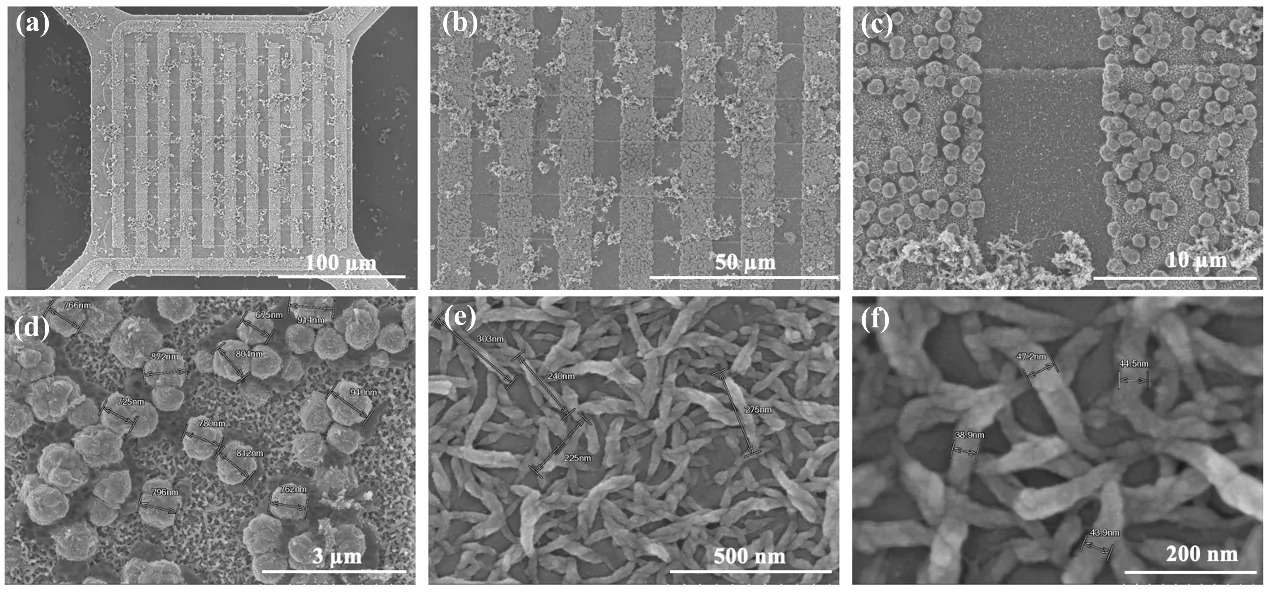
SEM images of the fabricated MEMS sensor: (**a**,**b**) overall morphology of the Au–PANI sensitive film; (**c**,**d**) detailed microstructural distribution of the Au microspheres in the sensitive layer; (**e**,**f**) highly magnified architectures of the in situ polymerized PANI nanorods.

The microstructural topographies of the Au–PANI MEMS sensors were systematically characterized utilizing FE-SEM, as comprehensively presented in Figure 1. A homogeneous and continuous sensing layer uniformly coats the sensor substrate in Figure 1a,b. At higher magnifications in Figure 1c,d, discrete submicron Au microspheres are densely distributed on the film surface. Their diameters range from 550 to 910 nm, with an average value of approximately 750 nm. Au microspheres exhibit an obvious preferential distribution over the interdigitated electrode (IDE) microheater regions rather than uniform random dispersion across the entire sensing film. This uneven distribution originates from site-selective electrodeposition. During the in situ growth of Au microspheres, conductive IDE fingers serve as preferential nucleation sites for Au^3+^ reduction, which accelerates the formation and deposition of Au microstructures on electrode areas [16]. As shown in Figure 1e,f, in situ oxidative polymerization of aniline directly on the MEMS platform produces tangled PANI nanorod networks. According to statistical analysis, the interconnected nanorods have lengths ranging from 220 to 310 nm (average of 250 nm) and diameters of 40–55 nm (average of 45 nm). The hierarchical network structure ensures favorable mechanical robustness and creates a highly porous framework with enlarged surface area. Such structure favors rapid trace gas transport and exposes abundant active adsorption sites, which is essential for achieving superior gas-sensing properties.

**Figure 2:**
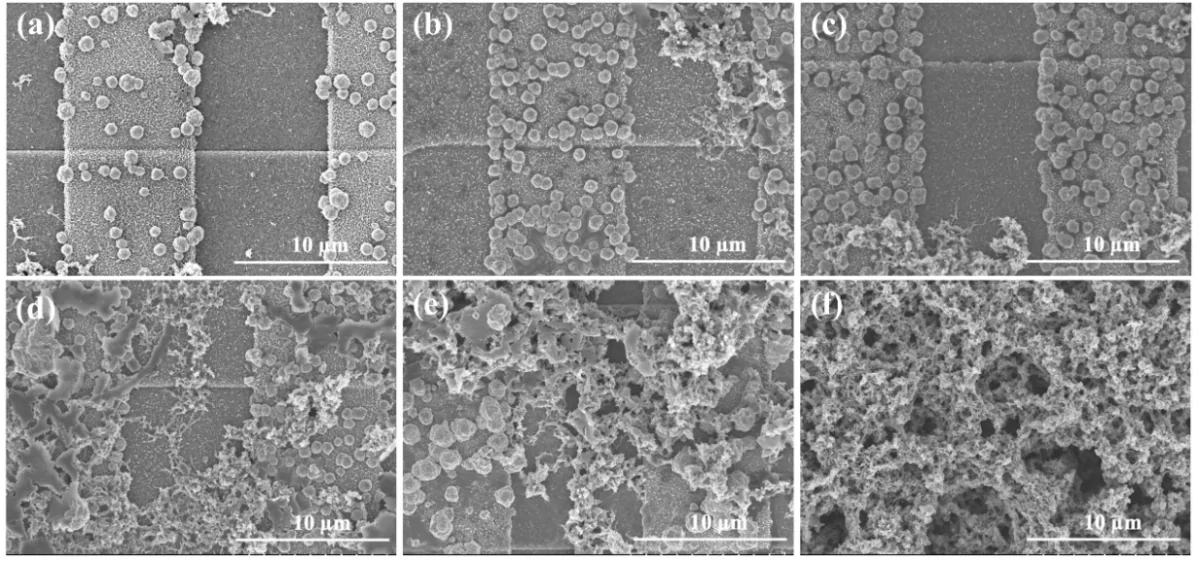
SEM micrographs of the Au–PANI sensor doped at various amounts of Au^3+^: (**a**) 0.001 M; (**b**) 0.002 M; (**c**) 0.004 M; (**d**) 0.008 M; (**e**) 0.01 M; and (**f**) 0.012 M.

Figure 2 displays the structural evolution and surface morphology of in situ polymerized Au–PANI nanocomposite films with varying Au^3+^ doping concentrations. At a low Au^3+^ concentration of 0.001 M (Figure 2a), discrete PANI domains scattered across the substrate are decorated with isolated Au microspheres. When the precursor concentration increases to 0.002 M and 0.004 M (Figure 2b,c), the number density and surface coverage of Au microspheres gradually rise. Meanwhile, the PANI film becomes visibly thicker, which is attributed to enhanced polymerization induced by sufficient metal ion oxidants [17].

Further increasing the Au^3+^ concentration to 0.008 M (Figure 2d) triggers a notable morphological transformation. The separated units interconnect with each other, forming an integrated and continuous porous network. Nevertheless, as the concentration reaches 0.012 M (Figure 2e,f), excessive loading of functional materials causes severe agglomeration. The film surface is densely packed, and the originally open porous channels are largely blocked by aggregated Au microspheres and polymer clusters. Such dense stacking reduces the available active surface area and hinders gas diffusion. These results reveal that the microstructure and pore distribution of Au–PANI sensing films can be effectively tuned by adjusting the initial Au^3+^ content. This structural regulation provides a solid basis for optimizing the overall gas-sensing performance.

**Figure 3:**
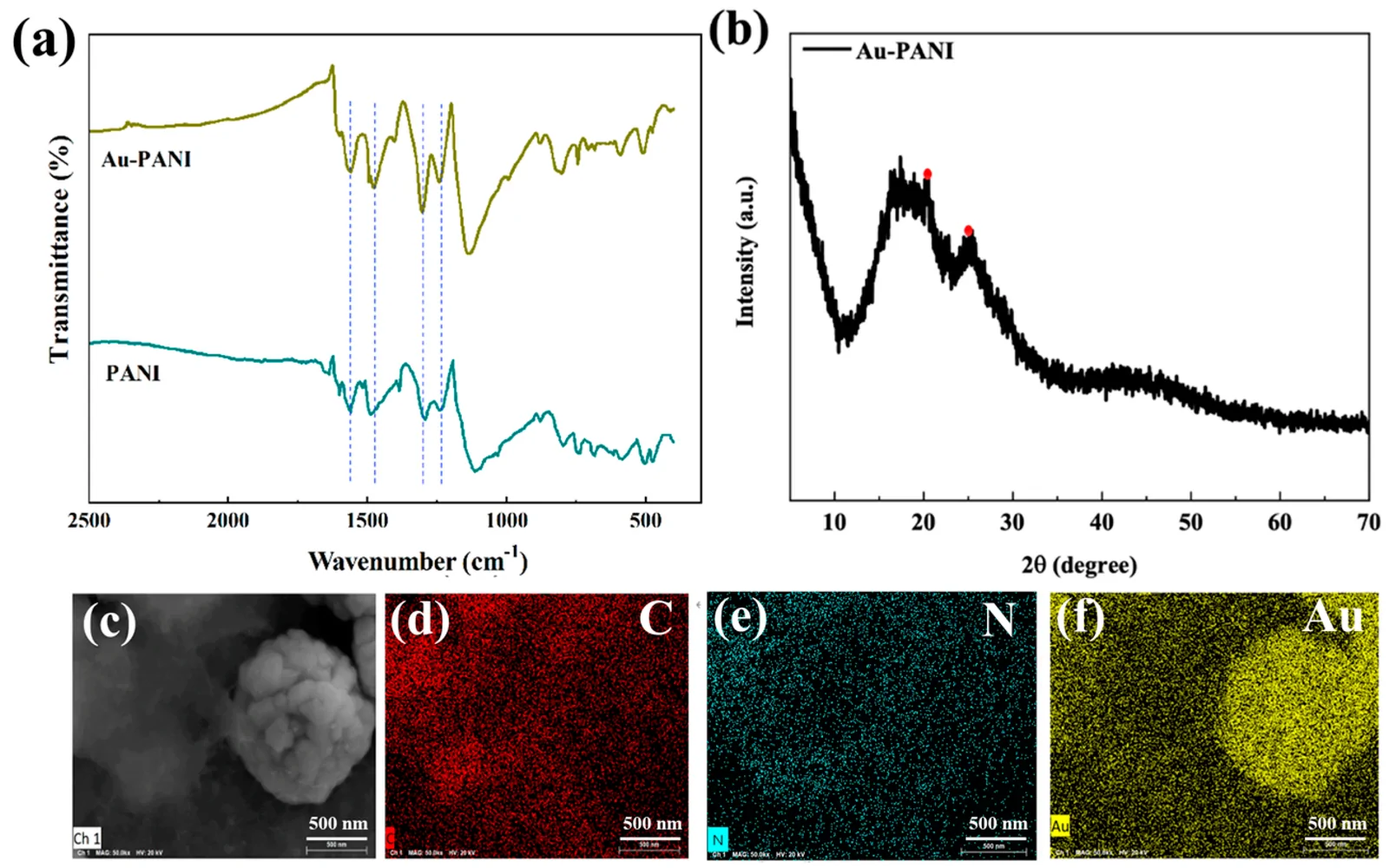
(**a**) FT-IR spectrum of the PANI and Au–PANI nanocomposite and (**b**) XRD pattern of the Au–PANI nanocomposite. (**c**) Corresponding SEM morphology, and (**d**–**f**) elemental maps for carbon (C), nitrogen (N) and gold (Au). (Scale bar = 500 μm).

Successful polymerization of the Au–PANI nanocomposite is confirmed using FT-IR spectroscopy, as shown in Figure 3a. Multiple characteristic absorption bands are observed in the Au–PANI spectrum. The prominent peaks at 1560 cm^−1^ and 1480 cm^−1^ are assigned to the C=C stretching vibration of quinoid rings and benzenoid rings, respectively. In addition, the characteristic bands at 1294 cm^−1^ and 1235 cm^−1^ originate from aromatic C–N stretching and delocalized C–N^+^ stretching modes of the PANI matrix [11,18]. For comparison, the FT-IR spectrum of pure PANI was acquired under identical conditions. Pure PANI exhibits the same characteristic quinoid/benzenoid and C–N bands, verifying that Au modification preserves the intrinsic PANI molecular backbone.

Furthermore, a complementary crystallographic evaluation is presented in Figure 3b. Two distinctive broad XRD peaks centered at 2θ values of around 20.5° and 23.9° are clearly observed in red dots, which are intrinsically attributed to the semi-crystalline structural nature of the PANI matrix [18,19]. No distinct diffraction peaks corresponding to Au are detected. XRD records averaged signals over the entire X-ray irradiated region. The low loading of crystalline Au limits the total volume of diffracting Au crystals and yields intensities below the instrument detection limit. The Au(111) diffraction position at ~38.2° 2θ falls on the slowly decaying broad background of semi-crystalline PANI, making weak Au signals indistinguishable. To further definitively confirm the presence and distribution of Au, EDS elemental mapping was conducted. Figure 3d–f displays the homogeneous distribution of C, N and Au elements over the PANI-Au composite particle, consistent with the morphology in the corresponding SEM image Figure 3c. Uniform overlap of carbon (d), nitrogen (e) and gold (f) signals verifies the successful incorporation of Au microspheres within the polyaniline matrix. Collectively, these crystallographic and spectroscopic empirical results solidly corroborate the successful synthesis of the Au–PANI nanocomposite.

**Figure 4:**
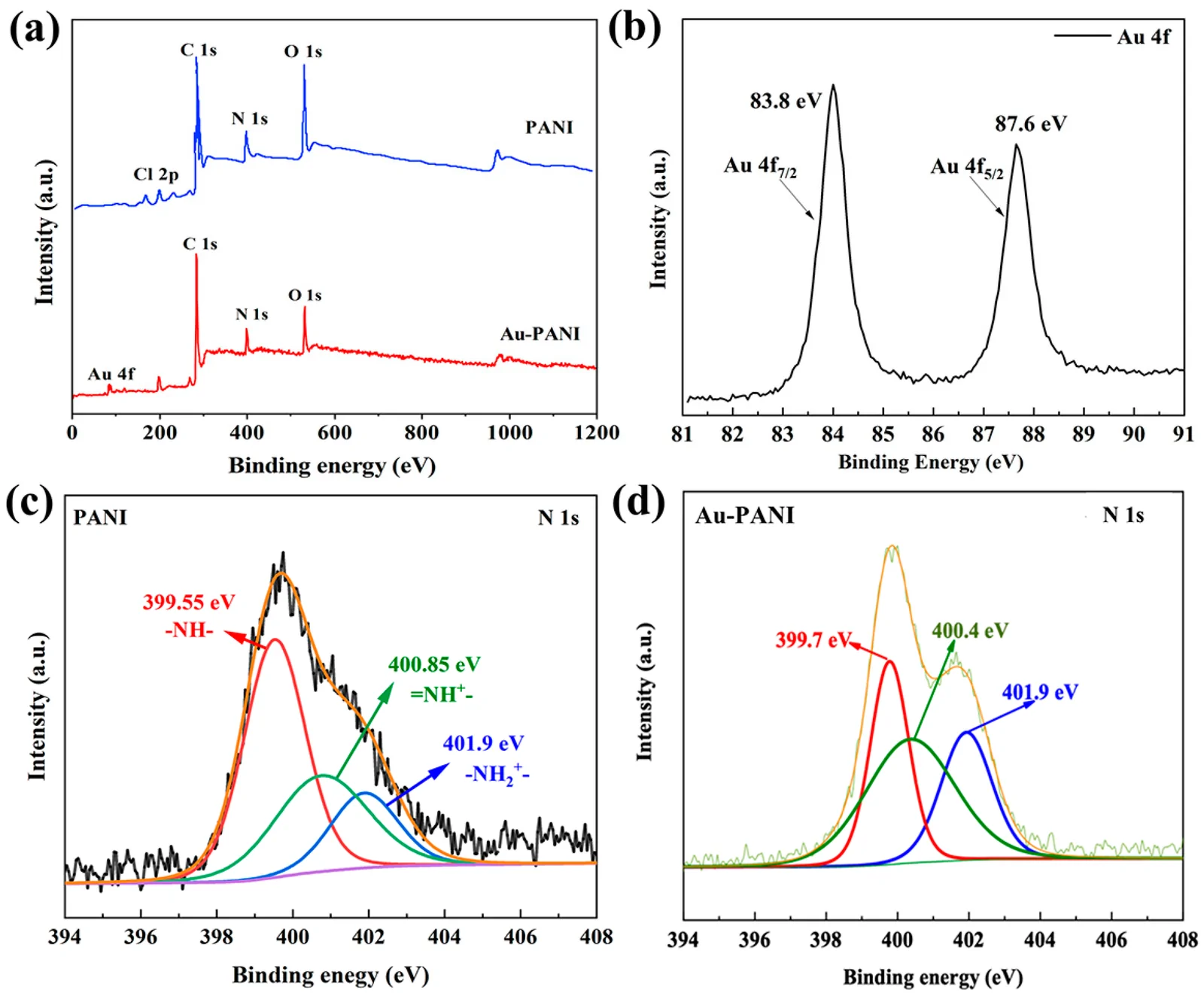
(**a**) XPS full survey spectrum of Au–PANI and PANI; high-resolution XPS spectra of (**b**) Au 4f for Au–PANI, (**c**) N 1s for pure PANI, and (**d**) N 1s for Au–PANI.

Figure 4 presents the XPS results of PANI and Au–PANI composites. For Au–PANI composites in Figure 4a, the full survey spectrum identifies C 1s, N 1s and O 1s signals originating from the PANI backbone, as well as Au 4f signals attributed to metallic Au. The high-resolution Au 4f spectrum in Figure 4b displays two characteristic spin–orbit split photoelectron peaks at 83.8 eV (Au 4f_7/2_) and 87.6 eV (Au 4f_5/2_), in good agreement with the standard binding energies of metallic Au. For pure PANI (Figure 4c), the high-resolution XPS spectrum can be deconvoluted into three distinct peaks corresponding to neutral –NH– (399.55 eV), protonated =NH^+^– (400.85 eV), and positively charged –NH_2_^+^– (401.90 eV) [10]. As shown in Figure 4d, when Au microspheres were incorporated into the PANI material, the binding energy of the peak of –NH– increased to 399.70 eV while the binding energy of the =NH^+^– peak fell to be 400.40 eV. These two competing binding energies suggest a significant chemical interaction or binding and/or some degree of electron transfer between Au and the nitrogen atoms on the PANI [20]. Also, as seen in Table 1, the amount of –NH– decreases from around 51% to around 40% in the composite, whereas the amount of =NH^+^– increases from about 31% to around 41% and –NH_2_^+^– remains relatively constant, at around 19%. As a consequence, there is an increased overall level of protonated sites to ~60% for the composite Au–PANI. The doping efficiency is enhanced by the existence of extra polaronic N^+^ species which further improves the NH_3_ gas sensing capabilities of the composite.

**Figure 5:**
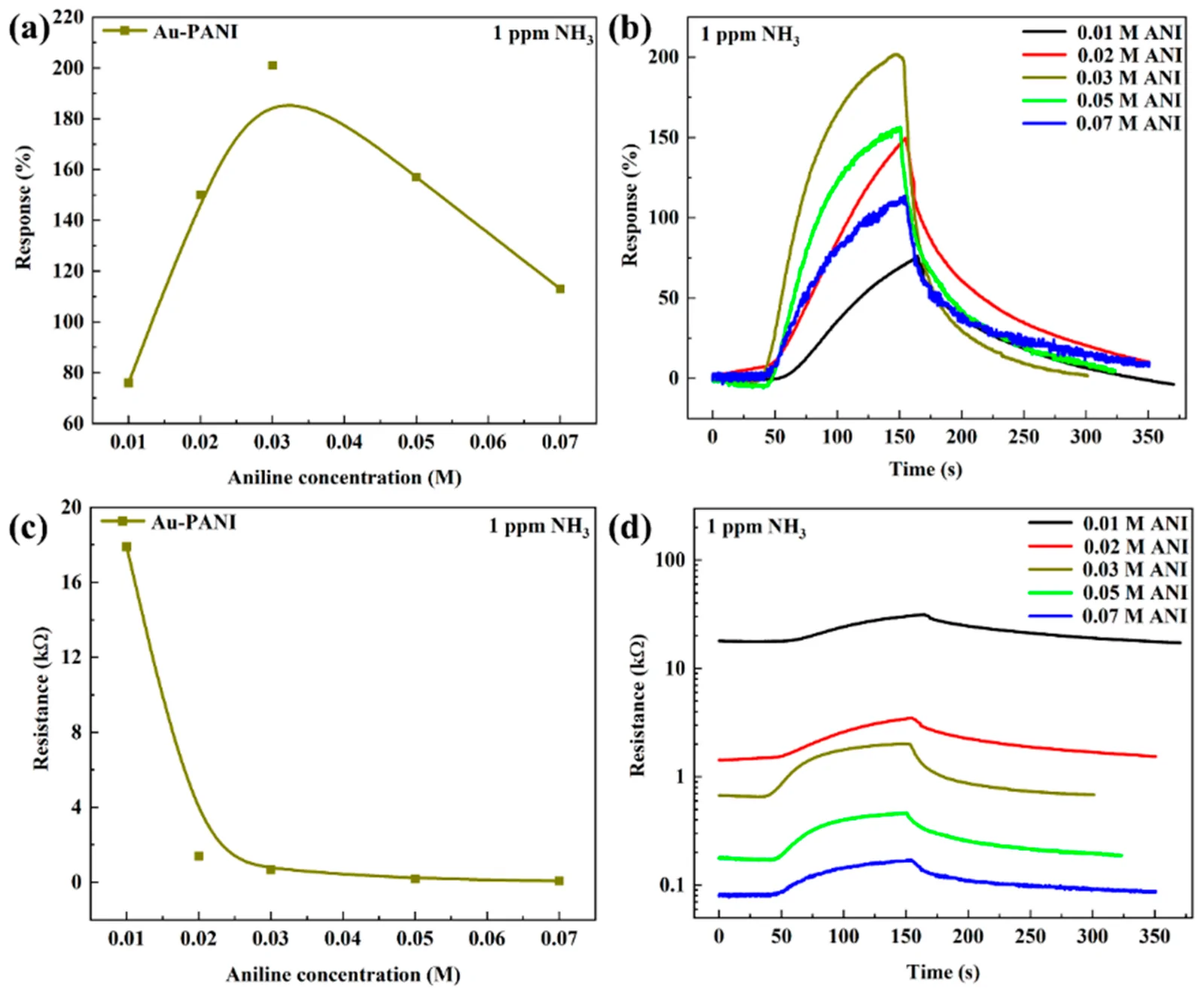
Response of Au–PANI MEMS sensors with different aniline concentrations (0.01–0.07 M) toward 1 ppm NH_3_. (**a**) Response variation against aniline concentration (optimal at 0.03 M); (**b**) dynamic response transients; (**c**) baseline resistance versus aniline concentration; (**d**) time-resolved resistance profiles during gas testing. (25 °C, 50% RH).

Figure 5a presents the response of the Au–PANI MEMS sensor to 1 ppm NH_3_ as a function of the aniline concentration (0.01–0.07 M). A clearly defined non-monotonic relationship is seen here. The peak in the sensor’s response occurs at 201% when the aniline concentration reaches its maximum value of 0.03 M. The associated transient response–recovery plots (Figure 5b) demonstrate that the best concentration for both sensitivity and reaction kinetics is achieved at the same point. As shown in Figure 5c, the baseline resistance decreases continuously from 17.9 kΩ down to 0.08 kΩ as the monomer concentration increases. It is evident in Figure 5c that the largest decrease occurs between 0.01 M and 0.02 M. The primary cause of this resistance drop is directly related to changes in the microstructure of the material as it undergoes in situ polymerization. The polymer structure becomes increasingly dense and thickened with increased aniline concentration, creating continuous pathways for electrical conduction by percolation. While the density increase also creates barriers to molecular diffusion of ammonia into deeper regions of the polymer matrix, leading to decreasing gas sensing responses above the optimal concentration.

Figure 5d shows the time-resolved resistance profiles for each composition which also indicate typical p-type semiconductor characteristics of the composite over all concentrations. The results show that the modulation of the aniline concentration can control both the morphology and the initial resistances, thus offering a reliable method by means of which one may adjust the gas-sensitivity of Au–PANI MEMS sensors.

**Figure 6:**
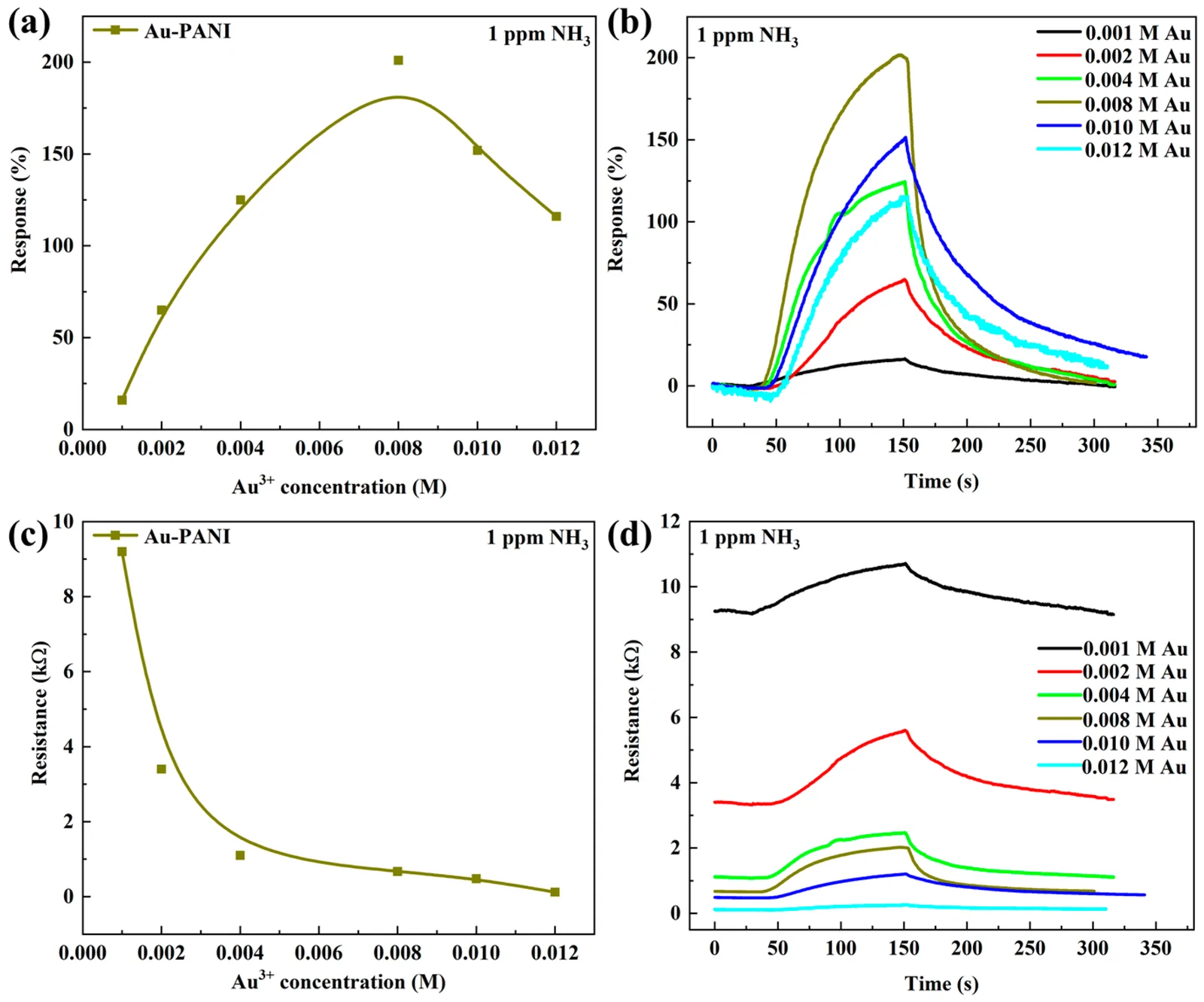
Response of Au–PANI MEMS sensors with different Au^3+^ concentrations (0.001–0.012 M) toward 1 ppm NH_3_. (**a**) Response variation against Au^3+^ concentration (optimal at 0.008 M); (**b**) dynamic response transients; (**c**) baseline resistance versus Au^3+^ concentration; (**d**) time-resolved resistance profiles during gas testing. (25 °C, 50% RH).

The influence of Au^3+^ doping concentration (0.001–0.012 M) on the performance of Au–PANI MEMS sensors has been thoroughly examined at 25 °C and 50% RH. As shown in Figure 6a, a non-linear increase in response towards 1 ppm of ammonia with an increase in the concentration of Au^3+^ doping. It increases from ~17% to a maximum of 201% at 0.008 M Au^3+^ and decreases at higher doses. From the transient response–recovery curves (Figure 6b), it is seen that the best reaction kinetics were shown at 0.008 M concentration. Meanwhile, Figure 6c shows that the baseline resistance is continually decreased from 9.2 kΩ to 0.12 kΩ as Au^3+^ concentration increased, with a sharp drop occurring below 0.004 M. The increase in conductivity is due to the increase in concentration of metallic gold microspheres in the PANI matrix which facilitates effective transport of charge carriers. More fundamentally, as further elaborated in the sensing mechanism section, the energy band alignment at the Au/PANI hetero-interface induces a spontaneous electron transfer from PANI to Au, creating a robust hole accumulation layer that drastically boosts the majority carrier concentration and accounts for this macroscopically diminished baseline resistance.

Furthermore, the time-resolved resistance profiles of Figure 6d show a distinct resistance increase upon NH_3_ exposure, verifying the characteristic p-type semiconducting behavior of the composite. In conclusion, the inkjet-printed Au–PANI sensor optimized with 0.03 M aniline and 0.008 M Au^3+^ delivers the best overall gas-sensing performance, demonstrating that precise dopant regulation is essential for optimizing composite-based MEMS devices.

**Figure 7:**
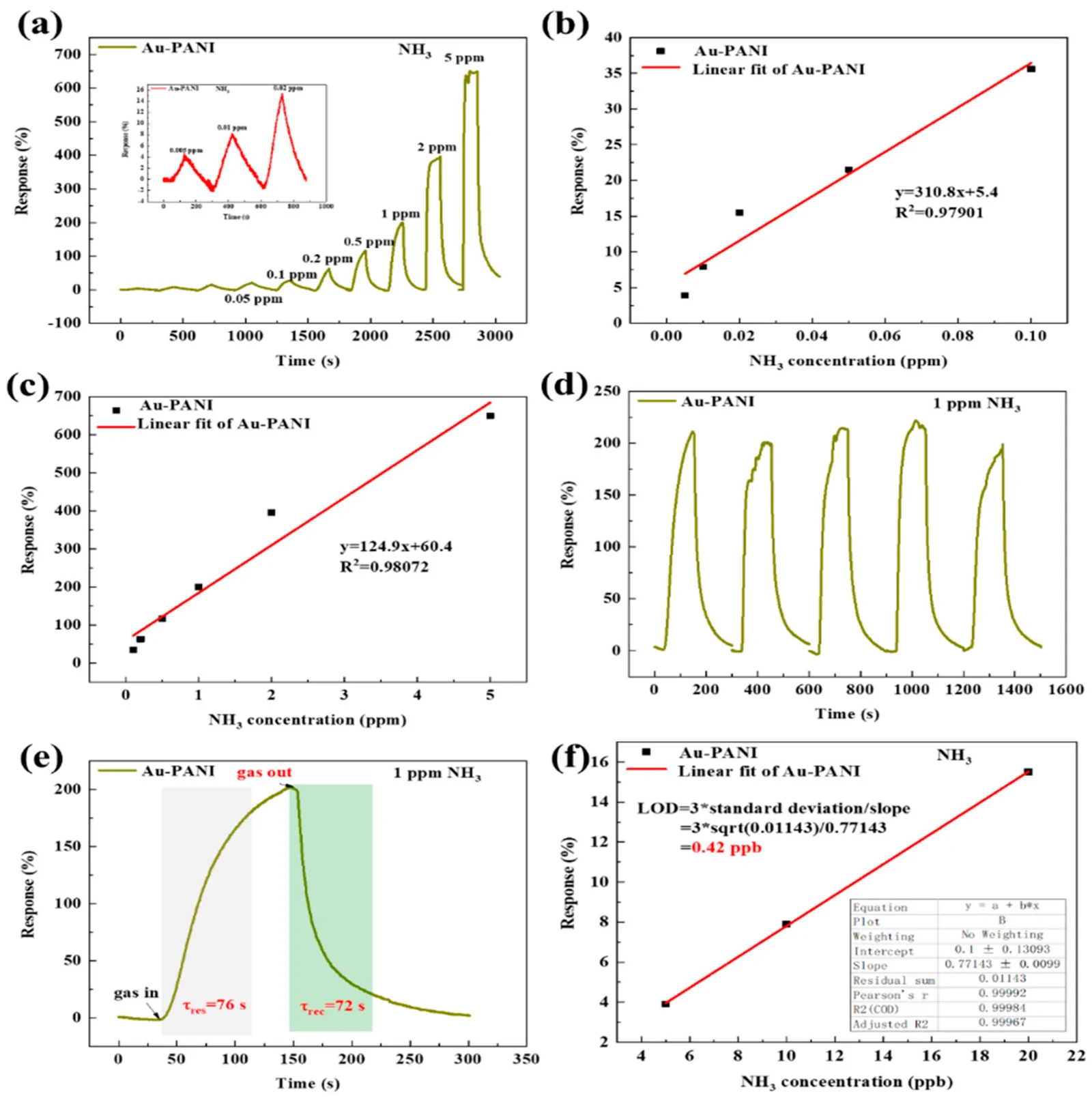
Comprehensive NH_3_ gas-sensing performance of the optimized Au–PANI MEMS sensor: (**a**) dynamic response transients to varying NH_3_ concentrations; linear calibration curves correlating the response magnitude and NH_3_ concentration within the (**b**) trace-level regime (0.005–0.1 ppm) and (**c**) ppm-level regime (0.1–5 ppm); (**d**) cyclic reproducibility over five continuous response–recovery cycles toward 1 ppm NH_3_; (**e**) specific response and recovery time toward 1 ppm NH_3_; and (**f**) extrapolated theoretical limit of detection (LOD) (25 °C and 50% RH).

The overarching gas-sensing performance of the Au–PANI MEMS sensor toward NH_3_ detection at 25 °C and 50% RH is comprehensively illustrated in Figure 7. Initially, the dynamic transient responses of the device toward varying NH_3_ concentrations (ranging extensively from 0.005 ppm to 5 ppm) were systematically investigated under standardized environmental conditions (25 °C and 50% RH), as depicted in Figure 7a. The inset shows the further characterization of the dynamic response at low NH_3_ concentrations of 0.005–0.02 ppm. The response increases substantially with ammonia concentration, from 4.2% (0.005 ppm) to 649.7% (5 ppm). Impressively, the Au–PANI MEMS device reaches a response of 201% to 1 ppm NH_3_. In comparison, pure PANI only attains 82%, representing over two times improvement.

The relationship between the goal NH_3_ concentration and the sensor’s response was thoroughly investigated statistically. Distinctive, well-defined linear regressions were evident in two distinct regimes of concentrations, as illustrated graphically in Figure 7b. Reliably correlation coefficients R^2^ values of 0.97901 and 0.98072 were identified within the ultra-low ppb regime (0.005 ppm to 0.1 ppm) and ppm regime (0.1 ppm to 5 ppm), respectively, as shown in Figure 7b,c. In addition to establishing reliable correlations in both regimes, an analytical sensitivity was established for each concentration regime; specifically, sensitivities of 310.8 and 124.9 were identified for the ppb and ppm regimes, respectively. A significantly greater sensitivity was demonstrated within the very low ppb regime relative to that observed within the ppm regime. The rationale for this concentration-dependent difference lies in the surface adsorption kinetics [21] as follows. At extremely low concentrations, there exist vast numbers of unoccupied adsorption sites on the surface compared to the number of NH_3_ molecules entering the solution. Conversely, at higher concentrations, many of these active sites are occupied, thereby resulting in a significant reduction in the stoichiometric ratio of available adsorption sites to target NH_3_ molecules [12]. Additionally, the high affinity interactions in the ppb regime are enhanced by the electronic and chemical sensitization of the gold microspheres, resulting in a significant decrease in the activation energy required for chemisorption at low gas coverages [22]. As previously explained, incorporating Au catalysts strategically not only amplifies total sensitivity but also significantly improves the linear correlation between response magnitude and NH_3_ concentration.

The cyclic reproducibility over five continuous response–recovery cycles for the Au–PANI MEMS ammonia gas sensor is shown in Figure 7d. The high level of cyclic reproducibility observed for this Au–PANI MEMS ammonia gas sensor after five consecutive response/recovery tests confirms its very stable operation with repeated exposures to 1 ppm NH_3_. In addition, as indicated in Figure 7e, the Au–PANI MEMS ammonia gas sensor exhibits fast response and recovery times to 1 ppm NH_3_ at room temperature and 50% RH of about 76 s and 72 s, respectively. Furthermore, a theoretically achievable LOD was estimated through signal/noise ratio extrapolation as shown in Figure 7f at 0.42 ppb which is significantly lower than the previously reported LOD of 19.6 ppb obtained from the pure PANI membrane-based sensor. These experimental findings validate that the selective incorporation of Au microspheres drastically lowers the detection threshold, hence leading to an exponential advancement in the trace-level detection capabilities of the sophisticated MEMS sensing platform.

**Figure 8:**
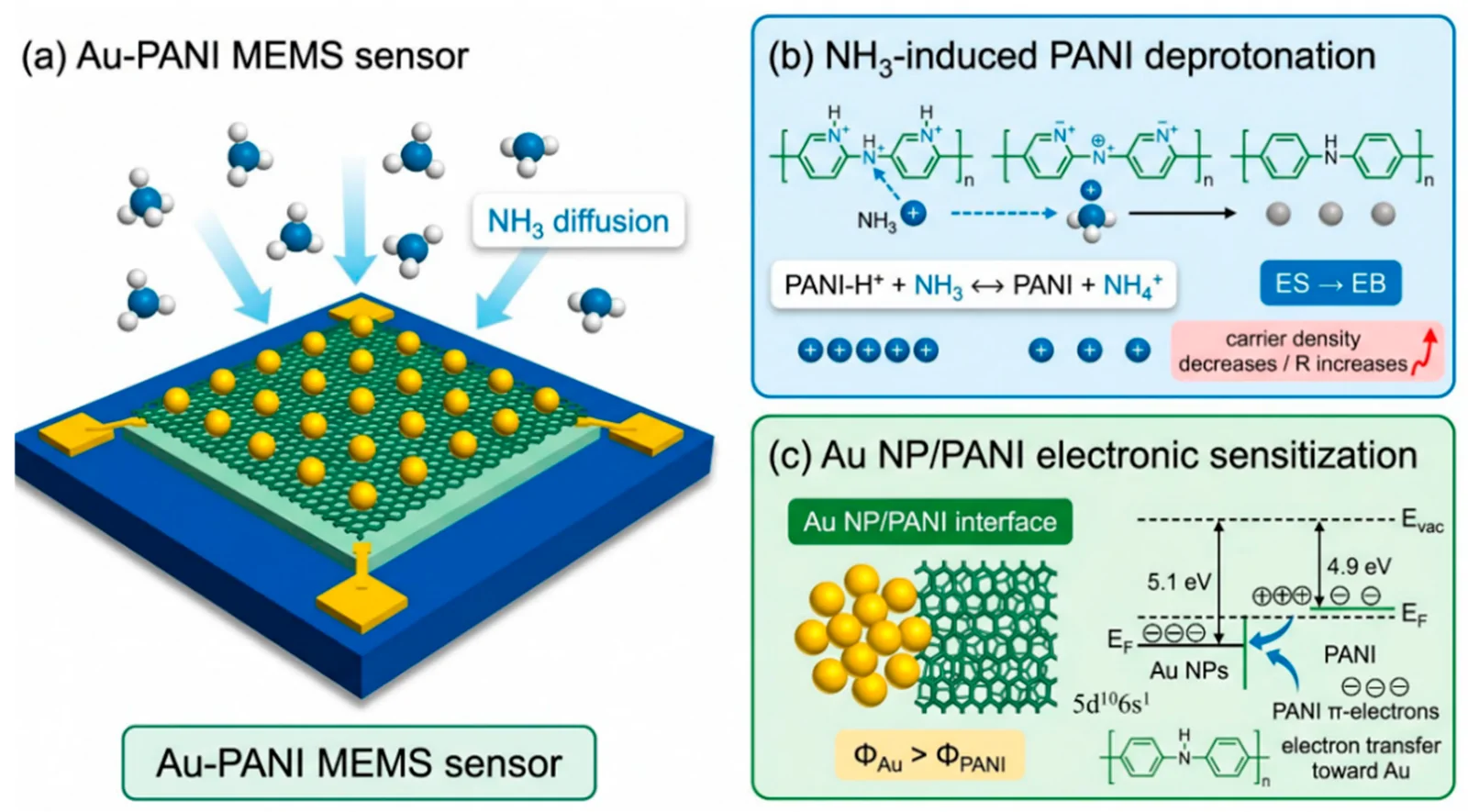
NH_3_ gas-sensing mechanism for the Au–PANI MEMS sensor: (**a**) representative model of NH_3_ gas diffusion and adsorption onto the hybrid sensing layer; (**b**) chemical mechanism for the NH_3_-induced deprotonation of PANI from emeraldine salt to emeraldine base; and (**c**) energy band diagram of electronic and chemical sensitization at the Au/PANI hetero-interface.

The operation mechanism of the Au–PANI MEMS gas sensor to detect ammonia is shown in Figure 8. It can be seen from Figure 8a that the structure of the sensor includes an optimum Au–PANI nanocomposite which allows for good NH_3_ diffusivity on the functionalized surface. Therefore, the basic function of the polyaniline base as a sensor is due to its ability to reversibly accept protons. Upon in situ chemical synthesis, the acidic environment formed by hydrochloric acid causes the polyaniline backbone to become protonated to produce the high-conductivity emeraldine salt (ES) form of polyaniline. This ES form of polyaniline provides the protonated nitrogen atoms (-NH^+^-) as active sites at which gas absorption occurs and also provides the pathways for electron conduction [11,23].

When exposed to NH_3_ molecules as shown in Figure 8b the lone pair electrons of the Lewis base NH_3_ interact with the positively charged nitrogen sites (protonated PANI − H^+^) to form NH_4_^+^ ions through the following reversible reaction:

(1) $$\mathrm{PANI}-H^{+}+{\mathrm{NH}}_{3}\;⇌\;\mathrm{PANI}+{{\mathrm{NH}}_{4}}^{+}$$

Deprotonation of this PANI structure leads to a structural phase transition from the high-conductivity ES to low-conductivity emeraldine base (EB). The decrease in hole carrier concentration in the PANI structure due to this phase transition causes an abrupt rise in the baseline resistances. When the target gas is purged and NH_3_ has desorbed from the surface, the matrix re-captures protons, which restores the initial conductive ES state and returns the resistance to its original level.

The improved room-temperature sensing properties, increased sensitivity, and faster response/recovery rates of the Au-modified PANI can be attributed to both electronic and chemical sensitivities as illustrated in Figure 8c, and therefore, based on energy considerations, PANI has a work function of Φ_PANI_ = 4.9 eV [24], while gold has an even larger work function of Φ_Au_ = 5.1 eV [25]. As such, when they come into close proximity with one another, there will be an immediate transfer of π electrons from the PANI’s conjugated backbone into the lower-lying unoccupied orbitals of the Au particles that will continue to occur until energy equilibrium is reached.

The area of electron loss around the Au microspheres creates a very dense layer of holes in the PANI that is very close to these Au microspheres. This means there are many more –NH^+^ bonding sites available for hydrogen than would be found without this layer. Additionally, the unique 5d^10^6s^1^ electronic configuration of the under-coordinated surface atoms on the Au NPs provides a catalytic spillover effect, reducing the adsorption energy barrier and accelerating gas interaction kinetics [26,27]. The combination of electronic sensitivity based on band structure and chemical activation through gold catalysis provides the best conditions for high NH_3_ adsorption rates and therefore optimal overall sensor performance.

**Figure 9:**
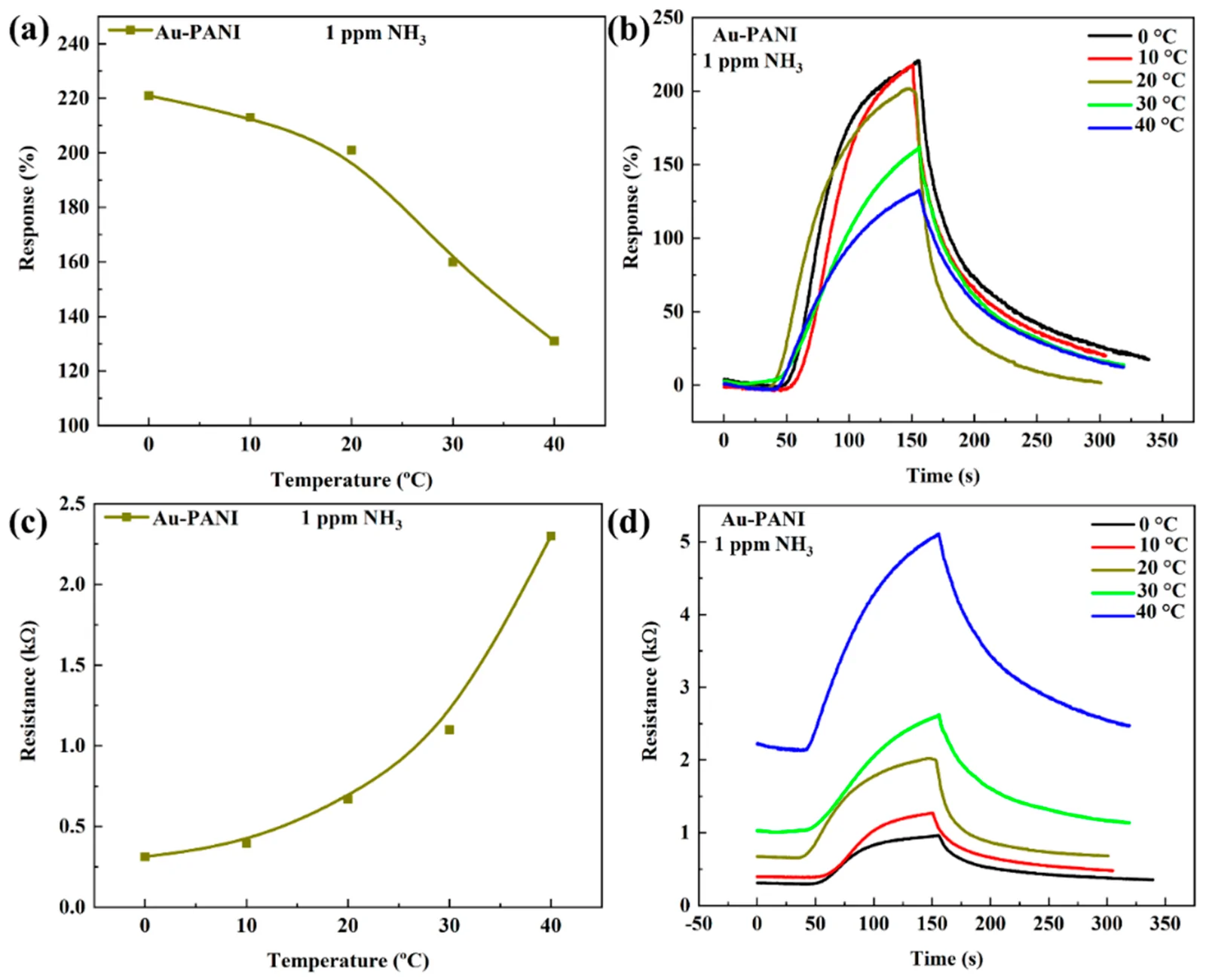
Temperature-dependent gas-sensing properties of the Au–PANI MEMS sensor: (**a**) sensing response toward 1 ppm NH_3_, (**b**) raw dynamic response curves, (**c**) baseline resistance at different temperatures, and (**d**) raw dynamic resistance curves.

The effects of temperature (0–40 °C) on the ammonia sensing properties of the Au–PANI MEMS sensor were investigated to assess the environmental stability of the device. As shown in Figure 9a, the sensor exhibits a negative correlation between temperature and its response toward 1 ppm NH_3_. The normalized responses at 0 °C, 10 °C, 20 °C, 30 °C, and 40 °C are 221%, 213%, 201%, 160%, and 131%, respectively, and gradually decrease with rising temperature. Raw dynamic response curves in Figure 9b further confirm that elevated temperatures reduce the peak response. These trends originate from the exothermic character of NH_3_ chemisorption on the Au–PANI surface. Increasing temperature shifts the adsorption equilibrium toward the desorbed state and reduces the population of NH_3_ bound to protonated sites, thus lowering the peak response. This behavior is governed by the adsorption isotherm and the temperature coefficient of the host–guest interaction, rather than full desorption under these moderate temperature conditions [24]. The results reveal that ambient temperature fluctuations significantly affect sensor output, which should be considered for practical field applications.

We further investigated the influence of temperature on the baseline resistance of the Au–PANI MEMS sensor. Distinct from the temperature-dependent sensing response originating from gas-surface interaction, the variation in baseline resistance reflects the intrinsic electrical properties of the PANI composite film. As displayed in Figure 9c, the baseline resistance rises markedly from approximately 0.3 kΩ to 2.3 kΩ over 0–40 °C. Using resistance values extracted from Figure 9c, the MEMS heater (~890 Ω at 25 °C) in series with a 5 kΩ load resistor consumes around 0.23 mW under a 3 V supply. The time-resolved resistance curves in Figure 9d intuitively illustrate such temperature-modulated resistance behavior during the whole adsorption–desorption cycle. This positive temperature coefficient can be explained by two factors: (i) elevated temperature promotes the desorption of adsorbed moisture, which serves as a temporary dopant for PANI and (ii) thermal expansion enlarges the distance between polymer chains, suppressing charge hopping transport. These thermal response characteristics lay a foundation for the development of temperature compensation algorithms to realize reliable trace NH_3_ detection under practical ambient conditions.

**Figure 10:**
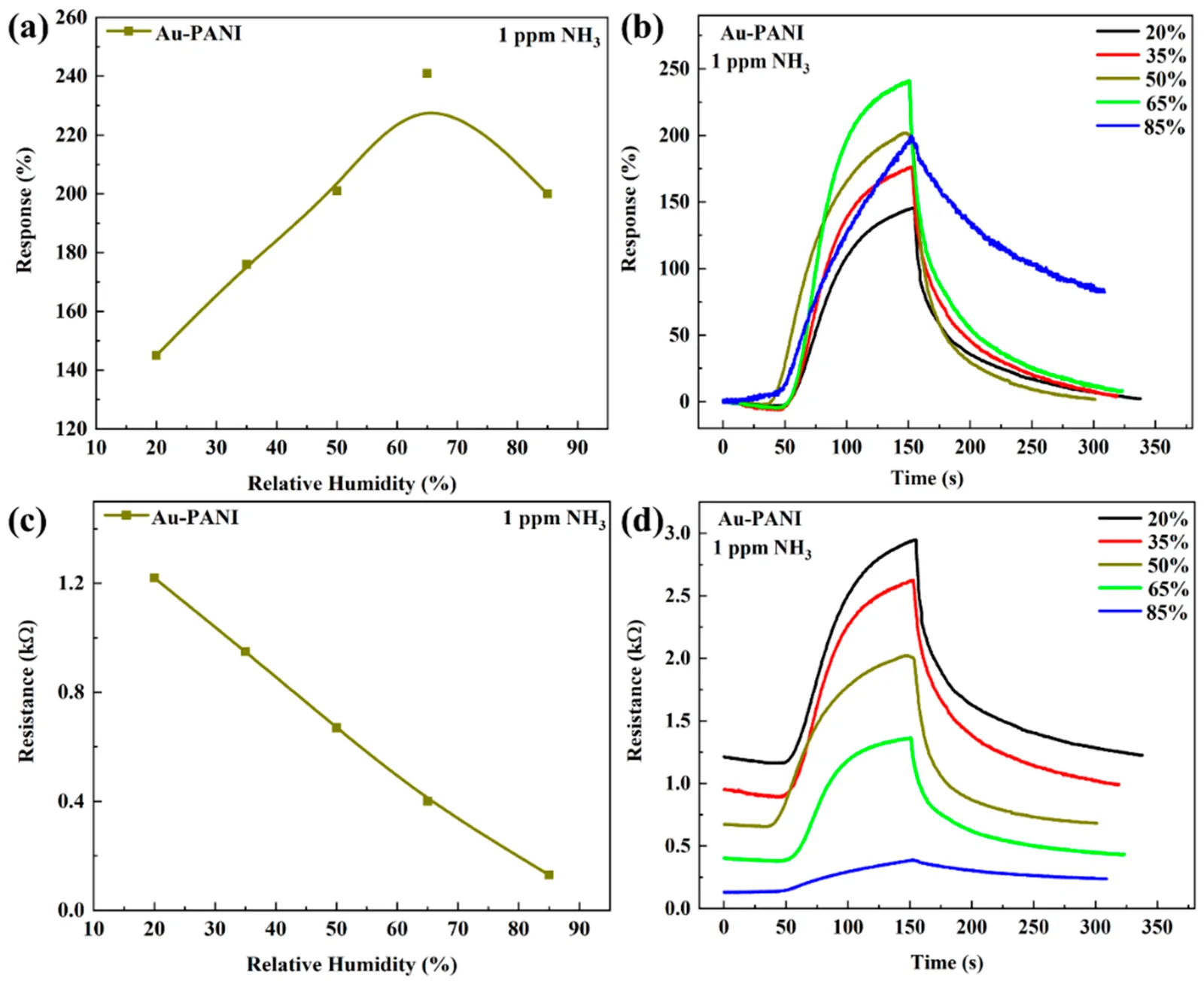
Humidity-dependent ammonia sensing performance of the Au–PANI MEMS sensor: (**a**) sensing response toward 1 ppm NH_3_, (**b**) raw dynamic response curves, (**c**) baseline resistance at different relative humidity levels, and (**d**) raw dynamic resistance profiles.

The effects of relative humidity (RH, 20–85%) on the sensing characteristics for ammonia with the Au–PANI MEMS Sensor are as follows and illustrated in Figure 10. In addition to illustrating the sensitivity towards ammonia at 1 ppm, it illustrates how the sensor’s response initially increased then decreased. This resulted in responses of 145%, 176%, 201%, 241%, and 200% towards 1 ppm NH_3_ under conditions of 20%, 35%, 50%, 65%, and 85% RH, respectively. The real-time transient curves in Figure 10b show similar results confirming the non-linear behavior as well as the maximum response amplitude occurring at approximately 65% RH. Moderate moisture facilitates matrix expansion, revealing additional protonated nitrogen centers (-NH^+^-) and aiding the ionic dissociation of NH_3_ into NH_4_^+^ and OH^−^, thus expediting the deprotonation process. However, at a high humidity of 85% RH, condensed water molecules create a screening layer that obstructs the active sites competitively.

A concurrent trend is presented by Figure 10c. The resistance baseline decreases from 1.22 kΩ at an initial RH of 20%, down to 0.13 kΩ when the RH has increased to 85%. This behavior results from the creation of a single, continuous layer of physisorbed water, which can activate the Grotthuss pathway for proton-hopping (H_3_O^+^), thus increasing the ionic conductivity significantly [28]. In addition, Figure 10c shows that with an excessively high humidity level of 85% RH, the baseline depression due to competitive water blocking in the substrate also causes significant contraction of the overall resistance changes as compared to those measured without water shielding, verifying the extent of analyte adsorption suppression [29]. These quantified dependencies offer critical guidelines for developing algorithmic humidity compensation models to secure reliable trace NH_3_ monitoring under fluctuating real-world ambient conditions.

**Figure 11:**
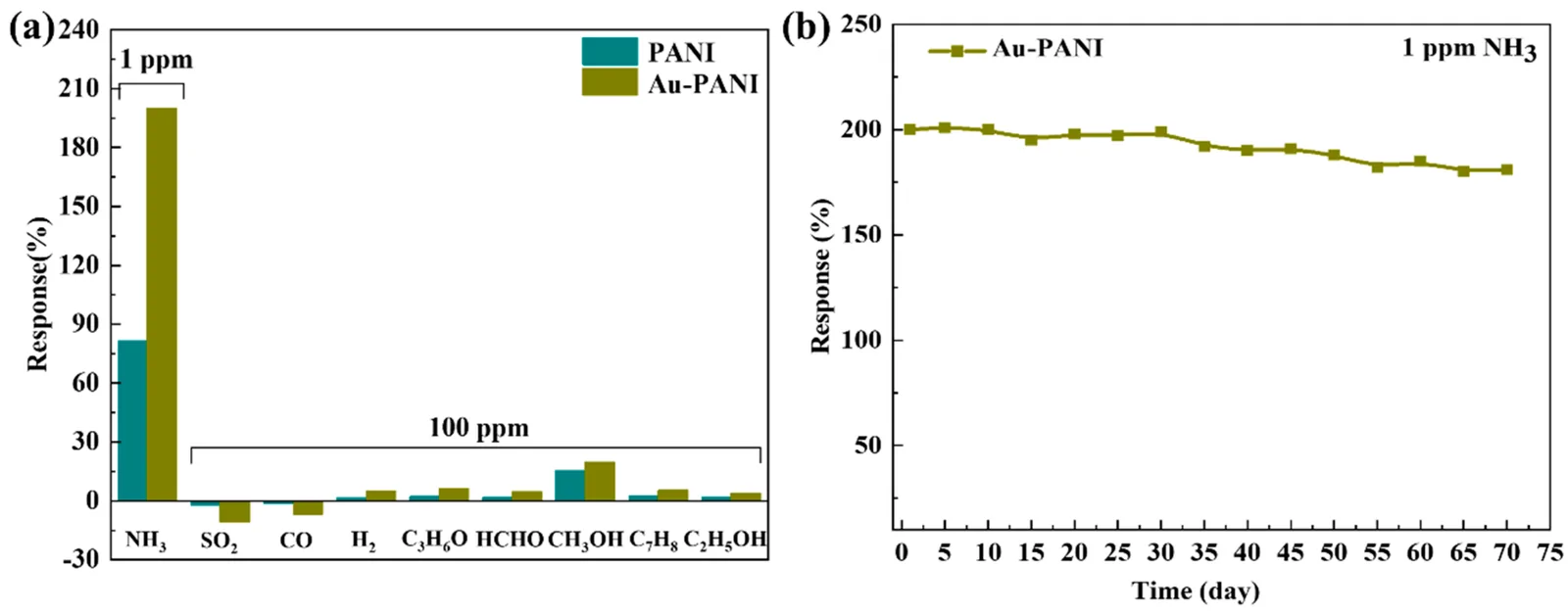
(**a**) Comparative cross-selectivity profiles of the pure PANI and Au–PANI MEMS sensors against nine distinct interfering gases; (**b**) long-term chronological stability of the optimized Au–PANI MEMS sensor toward 1 ppm NH_3_. (25 °C and 50% RH).

The cross-selectivity profiles of the pure PANI and Au–PANI MEMS sensors are evaluated in Figure 11a against 1 ppm NH_3_ and a 100-fold higher concentration (100 ppm) of eight common interferents (SO_2_, CO, H_2_, C_3_H_6_O, HCHO, CH_3_OH, C_7_H_8_, and C_2_H_5_OH). Pure PANI exhibits limited NH_3_ responsiveness (~82%) and visible cross-sensitivity. In contrast, the Au–PANI sensor delivers an outstanding response of 201% exclusively toward NH_3_, while yielding negligible responses of −10.5%, −6.7%, 4.9%, 6.1%, 4.7%, 19.7%, 5.3%, and 3.8% to the respective interferents. This superb specificity stems from the target-specific Lewis acid–base interaction between the basic NH_3_ molecules and the protonated sites of PANI, which is further synergistically amplified by the unique 5d^10^6s^1^ catalytic orbital alignment of Au microspheres that preferentially lowers the chemisorption activation energy for NH_3_ over other interfering analytes [30]. Meanwhile, NH_3_ is a polar molecule with a dipole moment of approximately 1.47 D. Its dipole facilitates strong oriented Lewis acid–base interactions with positively charged –NH^+^ sites. This interaction accounts for the high selectivity observed in Figure 11a.

Figure 11b illustrates the long-term operating reliability of the Au–PANI sensor towards 1 ppm NH_3_ at 70 days. The response has decreased from 201% to 181%, a remarkable retention in terms of response of 90%. This slight reduction in response was attributed to the time-dependent relaxation of PANI chain structure and the irreversible loss of partial surface active sites. Therefore, the excellent performance characteristics presented here verify that the Au–PANI MEMS sensor possesses both superior long-term reliability and interference rejection characteristics for use in environmentally related applications.

As listed in Table 2, our Au-decorated PANI MEMS sensor delivers an ultralow detection limit of 0.42 ppb and favorable response/recovery speed against 1 ppm NH_3_ when compared with previously reported PANI composite sensors. Combined with scalable inkjet printing preparation, room-temperature operation, robust selectivity and long-term stability, this sensor holds great potential for low-power, mass-producible trace ammonia monitoring.

## 4. Conclusions

In this study, we have developed a gas sensor for NH_3_ using an Au–PANI nanocomposite printed on a MEMS micro-hot plate. The characterization of the sample has shown a hierarchical structure formed by polyaniline nanorods covered with gold micro-spheres; they induce a 60% matrix protonation level through a strong electronic interaction. For optimization purposes, we used aniline at 0.03 M and Au^3+^ at 0.008 M. At optimized conditions, the device showed an increase in response of 201% to 1 ppm NH_3_—i.e., two times higher than the one observed for pure PANI—and it had fast kinetics (i.e., time to reach saturation = 76 s/72 s). Moreover, we calculated a theoretical detection limit of 0.42 ppb. This improvement is due to the combination of electronic sensitization of the interface which generates a hole accumulation layer and the chemical activation due to Au spillover during reversible deprotonation of emeraldine salt to base. We performed environmental testing in order to obtain both the monotonic inverse thermal relationship (from 0 °C to 40 °C) and the non-monotonic relationship between relative humidity (20–85%) and sensitivity. These relationships provide baseline modulating data for potential algorithms in order to compensate for environmental changes. Finally, the Au-decorated polyaniline on the MEMS platform shows good selectivity towards a hundredfold excess of interferents and maintains its sensitivity up to 70 days, retaining about 90% of the initial response. Notably, the sensor operates at room temperature without microheater activation, limiting power consumption to the µW–mW range from the readout circuit. Such low power demand makes the sensor promising for wearable and battery-powered trace-NH_3_ monitoring.

## Figures and Tables

**Table 1:** XPS atomic ratios of PANI and Au–PANI nanocomposite.

Sample	Chemical Group	Binding Energy (eV)	Area Fractions (%)
PANI	–NH–	399.55	51
	=NH^+^–	400.85	31
	–NH_2_^+^–	401.90	18
Au–PANI	–NH–	399.70	40
	=NH^+^–	400.40	41
	–NH_2_^+^–	401.90	19

**Table 2:** Performance comparison of representative NH_3_ gas sensors.

Sensor Material	Preparation Methods	Response Value	Response/Recovery Time	LOD	Ref.
PANI@Ag	In situ polymerization	77%/100 ppm	18 s/15 s	/	[31]
PANI/Au@SiO_2_	In situ polymerization + droplet evaporation self-assembly	248%/10 ppm	35 s/133 s	10 ppb	[8]
Au/PANI/WS_2_	Hydrothermal method	286%/100 ppm	24 s/26 s	13.8 ppb	[32]
PANI@Ag/Cu	In situ polymerization	86%/300 ppm	10 s/8 s	/	[33]
n-GaN-Au/PANI	MOCVD + in situ polymerization	195%/200 ppm	28 s/49 s	10 ppb	[34]
Pt-PANI	impregnation method	24.5%/20 ppm	210 s/238 s	20.3 ppb	[35]
Au-decorated PANI	Inkjet printing + in situ polymerization	201%/1 ppm	76 s/72 s	0.42 ppb	This work

## Data Availability

All data included in this study are available upon request by contacting the corresponding author.

## References

[1] Yoon G., Baek Y.E., Mirzaei A., Kim S.S., Lee Y., Kim J.-H. (2026). Interfacial amplification of NH_3_ sensing at low temperature using Au-decorated MoSe_2_/Ti_3_C_2_T_x_ MXene heterostructures. Appl. Surf. Sci..

[2] Meng F., Qi T., Zhang J., Zhu H., Yuan Z., Liu C., Qin W., Ding M. (2022). MoS_2_-Templated Porous Hollow MoO_3_ Microspheres for Highly Selective Ammonia Sensing via a Lewis Acid-Base Interaction. IEEE Trans. Ind. Electron..

[3] Ricci P.P., Gregory O.J. (2021). Sensors for the detection of ammonia as a potential biomarker for health screening. Sci. Rep..

[4] Wang J., Luo Y., Chen L.T., Su B.X., Ouyang Z.R., Ji Y.X., Zhou Z.Z., Xu T.L., Zhang X.J. (2025). Bioinspired Face Mask for Exhaled Breath Condensate Collection and Multiplexed Biomarkers Analysis. Acs Nano.

[5] Nijkoops A., Ciocca M., Aurora Costa Angeli M., Ahmad M., Boom R., Münzenrieder N., Lugli P., Petti L. (2026). Recent advances in conducting polymer chemiresistive sensors for ammonia gas detection: Materials, characterization, and applications. J. Phys. Mater..

[6] Kalaleh H.-A., Masri K., Allaf A.W., Alhabbal M.O., Mahmoud M. (2026). Polyaniline/reduced graphene oxide-NH_2_ hybrid for selective NH_3_ sensing. Mater. Chem. Phys..

[7] Shahdan D., Ahmad S., Chen R.S., Omar A., Zailan F.D., Abu Hassan N.A. (2020). Mechanical performance, heat transfer and conduction of ultrasonication treated polyaniline bio-based blends. Int. Commun. Heat Mass Transf..

[8] Chen G., Yuan Y., Lang M., Lv Z., Ma W., Gu N., Liu H., Fang J., Zhang H., Cheng Y. (2022). Core-shell Au@SiO_2_ nanocrystals doped PANI for highly sensitive, reproducible and flexible ammonia sensor at room temperature. Appl. Surf. Sci..

[9] Almaev A.V., Gaman V.I. (2017). Characteristics of Hydrogen Sensors Based on Thin Tin Dioxide Films Modified with Gold. Russ. Phys. J..

[10] Han D., Han X., Liu X., Zhang J., Zhao L., He X., Wang W., Xu B., Sang S. (2023). Conductometric gas sensor based on p-type GaN hexagonal pits /PANI for trace-level NH_3_ detection at room temperature. Sens. Actuators B Chem..

[11] Bekhoukh A., Moulefera I., Sabantina L., Benyoucef A. (2021). Development, Investigation, and Comparative Study of the Effects of Various Metal Oxides on Optical Electrochemical Properties Using a Doped PANI Matrix. Polymers.

[12] Kumar V., Patil V., Apte A., Harale N., Patil P., Kulkarni S. (2015). Ultrasensitive Gold Nanostar–Polyaniline Composite for Ammonia Gas Sensing. Langmuir.

[13] Petani L., Koker L., Herrmann J., Hagenmeyer V., Gengenbach U., Pylatiuk C. (2020). Recent Developments in Ozone Sensor Technology for Medical Applications. Micromachines.

[14] Lim S., Park S., Moon S.M., Yoo J., Choi J.H., Park J.H., Lee S., Min H., Kim Y.-T., Lee C. (2023). Paper-Based Inkjet-Printed Carbon Nanotube Colorimetric Chemiresistors for Detection of Chemical Warfare Agents. ACS Appl. Nano Mater..

[15] Zhang J., Zhang T., Song W., Yu R., Shen W. (2026). MEMS-based Au-PANI gas sensor with enhanced NH_3_ sensitivity and humidity tolerance at room temperature. Mater. Lett..

[16] Miao J., Lin J.Y.S. (2020). Nanometer-Thick Films of Aligned ZnO Nanowires Sensitized with Au Nanoparticles for Few-ppb-Level Acetylene Detection. ACS Appl. Nano Mater..

[17] Zhu K., Gao Y., Tan X., Chen C. (2016). Polyaniline-Modified Mg/Al Layered Double Hydroxide Composites and Their Application in Efficient Removal of Cr(VI). ACS Sustain. Chem. Eng..

[18] Hussin H., Gan S.-N., Phang S.-W. (2021). Development of water-based polyaniline sensor for hydrazine detection. Sens. Actuators A Phys..

[19] Ahmad H., BinSharfan I.I., Khan R.A., Alsalme A. (2020). 3D Nanoarchitecture of Polyaniline-MoS_2_ Hybrid Material for Hg(II) Adsorption Properties. Polymers.

[20] Tseng R.J., Baker C.O., Shedd B., Huang J., Kaner R.B., Ouyang J., Yang Y. (2007). Charge transfer effect in the polyaniline-gold nanoparticle memory system. Appl. Phys. Lett..

[21] Tai H., Jiang Y., Xie G., Yu J., Chen X. (2007). Fabrication and gas sensitivity of polyaniline–titanium dioxide nanocomposite thin film. Sens. Actuators B Chem..

[22] Li W., Wu X., Han N., Chen J., Tang W., Chen Y. (2016). Core-shell Au@ZnO nanoparticles derived from Au@MOF and their sub-ppm level acetone gas-sensing performance. Powder Technol..

[23] Almaev A.V., Kushnarev B.O., Yunin P.A., Korusenko P.M., Yakovlev N.N., Koroleva A.V., Zhizhin E.V., Yudin N.N. (2026). High-temperature CH_4_ sensors based on multilayer Ga_2_O_3_/SnO_2_/Ga_2_O_3_ thin-film structures with SiO_2_ barrier layers. Mater. Sci. Eng. B.

[24] Belabed C., Abdi A., Benabdelghani Z., Rekhila G., Etxeberria A., Trari M. (2013). Photoelectrochemical properties of doped polyaniline: Application to hydrogen photoproduction. Int. J. Hydrogen Energy.

[25] Gong H.R. (2009). The work function and electronic structure of coherent Ag–Au interfaces. Solid State Commun..

[26] Hong Y.-H., Chen Y.-S., Tsai M.-H., Lin H.-P. (2026). In-situ synthesis of Au/sulfonated electroactive polyimide nanocomposites and their application on catalytic reduction of 4-nitrophenol. Mater. Today Chem..

[27] Rogozhnikov N.A. (2020). A study of ammonia adsorption on gold face (111). Mater. Today Proc..

[28] Lee D.H., Kang H. (2021). Proton Transport and Related Chemical Processes of Ice. J. Phys. Chem. B.

[29] Song Y., Xia Y., Zhang W., Yu Y., Cui Y., Liu L., Zhang T., Liu S., Zhao H., Fei T. (2024). Humidity-Activated Ammonia Sensor Based on Carboxylic Functionalized Cross-Linked Hydrogel. Sensors.

[30] Song L., Tan K., Ye Y., Zhu B., Zhang S., Huang W. (2022). Amine-Functionalized Natural Halloysite Nanotubes Supported Metallic (Pd, Au, Ag) Nanoparticles and Their Catalytic Performance for Dehydrogenation of Formic Acid. Nanomaterials.

[31] Verma A., Kumar T. (2024). PANI@Ag nanocomposites gas sensors for rapid detection of ammonia. Polyhedron.

[32] Wang P., Tang C., Zhang L., Lu Y., Huang F. (2024). Hierarchical 0D/1D/2D Au/PANI/WS2 ternary nanocomposite NH_3_ sensor with high performance and fast response/recovery for food spoilage detection. Chem. Eng. J..

[33] Verma A., Kumar T. (2024). Ag/Cu doped polyaniline hybrid nanocomposite-based novel gas sensor for enhanced ammonia gas sensing performance at room temperature. RSC Adv..

[34] Han D., Wang Y., Wang Y., Duan Q., Li D., Ge Y., He X., Zhao L., Wang W., Sang S. (2024). Machine-learning-assisted n-GaN-Au/PANI gas sensor array for intelligent and ultra-accurate ammonia recognition. Chem. Eng. J..

[35] Qu Y., Ding Z., Lu X., Zhang F., Liu S., Liu J., Hou C., Li S. (2025). Pt-modified hollow tube-like polyaniline-based NH_3_ sensor. J. Hazard. Mater..

